# Toxicity Evaluation of a Polyphenolic Extract from *Flourensia cernua* DC through *Artemia* Lethality Assay, Hemolytic Activity, and Acute Oral Test

**DOI:** 10.1155/2024/2970470

**Published:** 2024-08-09

**Authors:** Yulma Lizbeth Aguirre-García, Ainara Castillo-Manzanares, Lissethe Palomo-Ligas, Juan Alberto Ascacio-Valdés, Lizeth Guadalupe Campos-Múzquiz, Sandra Cecilia Esparza-González, Raúl Rodríguez-Herrera, Sendar Daniel Nery-Flores

**Affiliations:** ^1^ School of Chemistry Autonomous University of Coahuila, Saltillo 25280, Coahuila, Mexico; ^2^ School of Dentistry Autonomous University of Coahuila, Saltillo 25125, Coahuila, Mexico

## Abstract

*Flourensia cernua* DC, commonly known as hojasen or tarbush, is a medicinal plant used in arid regions due to its therapeutic properties, especially in the treatment of gastrointestinal disorders. This study aimed to assess the toxicity of a polyphenolic extract obtained from *F. cernua*. This research involved both *in vitro* (hemolytic and brine shrimp assay) and *in vivo* tests (acute oral toxicity) to determine the safety profile of this extract. The extract was obtained through a novel ultrasound-microwave extraction and purified by ion-exchange chromatography. Analysis of the polyphenolic extract revealed a rich composition of flavonoids and hydroxycinnamic acids, mainly apigenin glycosides. In toxicity tests, the polyphenols did not exhibit toxicity towards *Artemia salina* at a concentration of 1 mg/ml. Furthermore, incubation at 500 *μ*g/ml for 4 hours showed a slight toxic effect on erythrocytes. In the acute oral toxicity test in mice, doses of 300 mg/kg and 2000 mg/kg did not result in animal mortality, indicating that the LD_50_ exceeds 2000 mg/kg. However, the higher dose induced signs of toxicity, including lethargy, drowsiness, piloerection, and a significant decrease in weight during the initial two days postadministration of the polyphenolic extract. In addition, histological analysis suggested potential kidney damage at the 2000 mg/kg dose. According to OECD guidelines, while the extract can be classified as category 5 (low acute toxicity) due to the absence of mortality at 2000 mg/kg, the observed signs of toxicity should be considered in the overall risk assessment. These findings highlight the potential of *F. cernua* in pharmaceutical and nutraceutical applications due to its high polyphenolic content. However, further investigations are necessary to explore the specific effects of the compounds present in the extract. In addition, continuous evaluation of its long-term toxicity is essential to fully understand the extract's safety profile and efficacy.

## 1. Introduction

The northern regions of Mexico, with their semiarid climate, host a diverse array of wild plants that thrive under extreme climatic conditions [1]. One such abundant plant in the Chihuahuan desert is *Flourensia cernua*. This region spans parts of Mexican states, Chihuahua, Coahuila, Durango, Zacatecas, San Luis Potosí, Nuevo León, and Tamaulipas, as well as Arizona, New Mexico, and western Texas in the United States of America [2]. *F. cernua,* commonly known as hojasen, tarbush, blackbrush, varnish bush, hojase, hoja ancha, and arbusto de alquitrán. It is a shrub characterized by its light brown or gray stem, which typically grows around 1 meter in height [3, 4]. The shrub is highly branched, resinous, and emits a distinctive tar-or hop-like odor. Its leaves are alternate, ovate to oval, and dark green [5, 6]. The plant bears pendulous flowers containing several yellow disc florets, and its fruit is a hairy achene, measuring up to 1 cm in length [7].

In Mexican markets, *F. cernua* leaves and flowers are commercially available for preparing infusions and are used in traditional medicine. Notably, *F. cernua* leaves have been used as a remedy for various gastrointestinal ailments, such as diarrhea, indigestion, and stomach pain. The plant has a history of traditional use because of its purgative, astringent, expectorant, and antirheumatic effects [5, 8, 9]. The phytochemical composition of *F. cernua* includes an array of polyphenolic compounds and essential oils. Mainly, flavonoids, such as flavones, flavanones, and flavonols, have been identified as the main constituents [10, 11]. The tarbush resin contains sesquiterpenes, acetylenes, p-acetophenones, benzofurans, and benzopyrans. Recent studies have explored the biological properties of the plant, and its extracts have been associated with antioxidant, anti-inflammatory, anticancer, and antidiabetic activities *in vitro* [12, 13]. Given its potential as a source of pharmacologically active compounds, evaluating the safety of its constituents is crucial. Some studies have reported the toxicity of *F. cernua*; its dry fruits have been described as toxic when consumed at approximately 1% of the animal's body weight, potentially leading to fatalities within the first 24 hours [14]. Chronic consumption of its leaves has been associated with liver damage, evidenced by elevated levels of serum gamma-glutamyl transpeptidase and aspartate aminotransferase activities, as well as hepatocytes apoptosis in ewe lambs [15]. Furthermore, the administration of a single 2000 mg/kg oral dose of tarbush extract resulted in signs of toxicity such as lethargy and drowsiness in rats within half an hour [16]. Therefore, this study aims to identify and quantify the polyphenol content in a hydroethanolic extract of *F. cernua* leaves. It also seeks to evaluate its toxicity using brine shrimp, hemolytic activity, and acute oral toxicity tests in mice.

## 2. Materials and Methods

### 2.1. Vegetal Material

**Figure 1:**
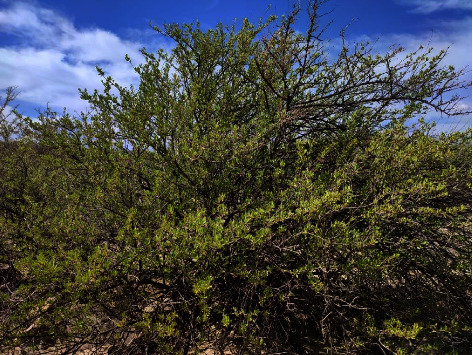
*Flourensia cernua* DC shrub.

*Flourensia cernua* DC samples were collected from the “La Angostura” region in Coahuila de Zaragoza (latitude: 25.30909 and longitude: -101.07395) in August. Random bushes were selected, and their branches were pruned (Figure 1). A specimen was pressed and prepared for identification at the ANSM herbarium, located in the Department of Botany at the Antonio Narro Agrarian Autonomous University. Professor José A. Villarreal Quintanilla assigned it the registration number 104702. The leaves were then air-dried at room temperature (22–26°C) for 72 h. After drying, the branches were separated, leaving only the leaves for further processing. Subsequently, the leaves were ground and sieved using a RO-TAP (Test Sieve Shaker, W.S. Tyler™), and particles smaller than 0.6 mm were collected.

### 2.2. Extraction of Phytochemical Compounds

A hydroethanolic extract of *F. cernua* leaves was obtained using a mass: volume ratio of 1 : 12. Initially, 83.33 g of the dry sample was weighed and mixed with 1 L of 30% ethanol. The mixture was then subjected to an ultrasonic-microwave (US-MW) reaction system (Nanjing ATPIO Instruments Manufacture Co. Ltd., Nanjing, China) under the conditions described in previous research [17]. The resulting hydroethanolic extract was filtered three times using filter paper (Whatman No. 41) to remove insoluble material and then stored at 4°C in amber containers.

### 2.3. Purification of Polyphenolic Compounds

After extract preparation, polyphenols were purified using ion-exchange chromatography, employing AmberLite XAD-16 (MilliporeSigma) as the stationary phase and a mobile phase consisting of water followed by 96% ethanol. Initially, water was passed through the column to wash away nonpolyphenolic compounds. Then, 96% ethanol was used as the desorption solvent to elute the polyphenols from the AmberLite resin. The ethanolic solution was collected in an Erlenmeyer flask until the mobile phase no longer exhibited color, indicating that the elution of polyphenols was complete. A 1 : 1 ratio of ethanol to extract volume was used to ensure complete elution of the polyphenols. Subsequently, the drying process was conducted in an oven set at 50°C for 24 h to evaporate the ethanol. Once dried, the polyphenols were scraped with a stainless-steel spatula to recover the powder, which was then weighed and stored in amber bottles at room temperature [18].

### 2.4. Determination of Total Hydrolyzable Polyphenols

To quantify the total hydrolyzable polyphenols, the Folin–Ciocalteu method was employed [19]. A 15 *μ*l sample of the polyphenol fraction at a concentration of 0.5 mg/ml (dissolved in 96% ethanol) was placed and mixed with 170 *μ*l of Milli-Q water in a 96-well microplate in triplicate. Next, 12 *μ*l of Folin–Ciocalteu reagent (Millipore) was added, and the mixture was incubated in the dark for 5 min at room temperature. Subsequently, 30 *μ*l of Na_2_CO_3_ (200 g/L) was added, and the solution was further incubated in the dark for one hour at room temperature. Finally, 73 *μ*l of water was added, and the absorbance was read at 765 nm using a spectrophotometer (Epoch BioTek ELISA plate reader). A calibration curve with gallic acid (Sigma-Aldrich; G7384) was used as the reference standard. The results were expressed as milligrams of gallic acid equivalent per gram of dry extract (mg GAE/g).

### 2.5. Determination of Condensed Polyphenols

The quantification of condensed polyphenols was performed using the HCl-butanol method [18]. In a 1.5 ml microtube, 167 *μ*l of the sample (1 mg/ml), 1 ml of HCl-butanol (1 : 9), and 33 *μ*l of ferric reagent (2% ferric ammonium sulfate in 2 N HCl) were combined. The mixture was then heated at 100°C in a digital dry bath (Labnet D1100) for 1 h. After cooling, 300 *μ*l from each tube was transferred to a 96-microplate well in triplicate. Finally, the absorbance at 460 nm was measured using a spectrophotometer (Epoch BioTek ELISA plate reader). Results were expressed as milligrams of catechin equivalent per gram of dry extract (mg CE/g) based on a calibration curve with catechin (Sigma-Aldrich; C1251).

### 2.6. Determination of Flavonoid Content

The quantification of flavonoids was conducted based on their reaction with aluminum ions in a basic medium [20]. In a 1.5 ml microtube, 200 *μ*l of the sample (1 mg/ml) was mixed with 280 *μ*l of Milli-Q water and 60 *μ*l of 5% NaNO_2_, and the resulting mixture was thoroughly blended. The solution was then incubated for 5 min at room temperature. Afterwards, 60 *μ*l of 10% AlCl_3_ was added, mixed, and incubated for 10 min at room temperature. Finally, 400 *μ*l of 1 M NaOH was added to the mixture. From this, 200 *μ*l was placed in a 96-microplate well in triplicate. The absorbance was read at 510 nm using a spectrophotometer (Epoch BioTek ELISA plate reader). The results were expressed as milligrams of catechin equivalent per gram of dry extract (mg CE/g) based on a calibration curve with catechin (Sigma-Aldrich; C1251).

### 2.7. Identification of Polyphenolic Compounds by HPLC/MS

Characterization of the *F. cernua* polyphenolic extract was carried out using reversed-phase high-performance liquid chromatography (HPLC) with a diode array detector (280 nm) and a mass spectrometer (MS) equipped with a liquid chromatography ion trap (Varian 500-MS IT; Agilent, Santa Clara, CA, USA) and an electrospray ion source. One milligram of the sample was diluted in 1 ml of 96% ethanol, filtered through a 0.45 *μ*m nylon membrane, and 5 *μ*l were injected into a Denali C18 column (3 *μ*m, 150 mm × 2.1 mm; Grace, Albany, OR, USA) maintained at 30°C. Eluents consisted of formic acid (0.2%, v/v; solvent A) and acetonitrile (solvent B). The elution conditions were in accordance with a previous study [21]. Mass spectrometer experiments were performed in negative mode [M-H]^−1^. Data were collected and processed using the MS Workstation software (version 6.9; Agilent, Santa Clara, CA, USA). The samples were first analyzed in a full scan mode acquired in the m/*z* range 50–2000, and further analyses were performed on a series of selected precursor ions. The HPLC/MS analyses were conducted using a bioactive compounds database (WorkStation version 2.0 database; Varian, Palo Alto, CA, USA). To estimate the relative content of the identified polyphenols, the area of each peak was calculated using the software GraphPad Prism 6.

### 2.8. Toxicity in Brine Shrimp

The toxicity test in *Artemia salina* was conducted following previously reported methods [22]. Initially, 83 mg of brine shrimp cysts were placed in 500 ml of simulated seawater (Supplementary Table 1) and kept at 26°C with intense light (5500 lux) in an artificial climate chamber (ECOSHEL C268D) for 24 h to promote cyst hatching. For the assay, 1900 *μ*l of simulated seawater was dispensed into each well in a 24-well microplate and 10 brine shrimp nauplii were added to each well. Subsequently, 100 *μ*l of the polyphenolic extract of *F. cernua* was placed at 5 different concentrations, 50, 100, 200, 500, and 1000 *μ*g/ml, in triplicate. As a positive toxicity control, potassium dichromate (K_2_Cr_2_O_7_) was used at a concentration of 200 *μ*g/ml. Polyphenolic extracts at different concentrations were prepared using a vehicle composed of 5% Tween 80, 5% DMSO, and 90% phosphate-buffered saline (PBS). In addition, a negative control with simulated seawater and a vehicle control were included. Following the addition of treatments, the brine shrimp nauplii were incubated for 24 h at 26°C in the dark. After incubation, the brine shrimp nauplii in each well were counted, considering a brine shrimp dead if it remained motionless for 10 s. Finally, the percentage of mortality for each treatment was calculated according to the following equation:

(1) $$\begin{array}{c} \%\;m\text{ortality}=\left(\frac{\text{total}\;Artemia\;salina\;-\;\text{living}\;Artemia\;salina}{\text{total}\;Artemia\;salina}\right)*100. \end{array}$$

### 2.9. Hemolysis Assay

The hemolysis assay was performed on two types of blood samples: mouse blood and human blood. The mouse blood sample was obtained from female mice of the BALB/*c* strain, acquired from the School of Chemistry, Autonomous University of Coahuila. The blood from mice was collected by intracardiac puncture using an EDTA (10%)-impregnated syringe with a 27G × 13 mm needle. Before the procedure, all animals were anesthetized with pentobarbital (150 mg/kg). The human blood sample was obtained from a healthy volunteer with no previous medical history of chronic diseases. Phlebotomy was performed on the peripheral veins of the arm, using a Vacutainer® 21G × 38 mm needle and a BD Vacutainer® K2 EDTA tube. After collection, the blood samples were centrifuged at 600 *g* for 5 min. A line was marked on the tube at the same height as the supernatant, which was then removed. The same volume of PBS solution was added to the erythrocyte suspension, carefully mixed, and centrifuged again at 600 *g* for 5 min. The supernatant was removed, and the washing process with the PBS solution was repeated twice. Once the washing steps were completed, the erythrocytes were suspended in PBS at a concentration of 2% (v/v). For this, 10 *μ*l of the erythrocyte pellet was diluted in 480 *μ*l of PBS with 10 *μ*l of the polyphenolic extract of *F. cernua* at different concentrations (100, 200, and 500 *μ*g/ml). The polyphenolic powder was dissolved in two vehicles as follows: (a) 5% Tween 80, 5% DMSO, and 90% PBS and (b) 25% DMSO and 75% PBS. The samples were then incubated at 37°C for 1, 2, 3, and 4 h. After incubation, the samples were centrifuged at 2000 *g* for 5 min and 100 *μ*l of the supernatant was placed in a well of the 96-well microplate in triplicate. Finally, the samples were read at a wavelength of 540 nm using a spectrophotometer Epoch BioTek ELISA plate reader. PBS and distilled water were used as negative and positive controls, respectively. The equation used to calculate the percentage of hemolysis was as follows:

(2) $$\begin{array}{c} \%\text{ hemolysis}=\left(\frac{{\text{sample}\;\text{Abs}}_{540}-\;\text{blank}\;\left(\text{PBS}\right){\text{Abs}}_{540}}{\text{total}\;\text{lysis}\;\left(dH_{2}O\right){\text{Abs}}_{540}-\text{ blank }\left(\text{PBS}\right){\text{Abs}}_{540}}\right)*100. \end{array}$$

### 2.10. Acute Oral Toxicity According to OECD 423

The study was performed after getting approval from the Ethics Committee of the School of Chemistry, Autonomous University of Coahuila with the registration number: P-FCQ-A-25-01-23-1. The acute oral toxicity test was performed following the guidelines of the OECD (Organization for Economic Co-operation and Development) 423 assay. Female mice (*Mus musculus*) of the BALB/*c* strain, weighing between 18 and 22 g and aged 8 to 12 weeks, were used for the study. According to the OECD guidelines, females are normally used because they generally exhibit slightly higher sensitivity to toxic effects. This ensures a more sensitive and reliable assessment of the potential toxicity of the substance being tested. Animals were housed and handled in accordance with the National Institutes of Health Guide for the Care and Use of Laboratory Animals (NIH publication no. 80-23, revised 1978) and the Mexican normative NOM-062-ZOO-1999. Mice were kept under controlled conditions with 12/12 h light/dark cycles, 22 ± 2°C, 40–60% relative humidity, with water and food (LabDiet 5001 Rodent Diet) available *ad libitum*.

Before the experiment, the mice were habituated in individual cages for one week. The assay began with a test dose of 300 mg/kg of the polyphenolic extract of *F. cernua* (*n* = 6); this is due to OECD guidelines 423, since it is recommended to use the starting dose of 300 mg/kg of body weight when there is no information about the substance to be tested. Mice were fasted for 4 h, allowing only access to water before administering the polyphenolic extract. Then, the extract, dissolved in the vehicle (5% DMSO, 5% Tween 80, and 90% PBS), was administered orally with an oral gavage needle (20G × 38 mm). After that, the food was removed for an additional 2 h. Animals were observed periodically during the first 24 hours, with particular attention given to the first 4 hours, and then daily for a total observation period of 14 days. Mice were weighed daily throughout this period. If no mortality was observed in mice treated with the 300 mg/kg dose, an additional group of mice was used at a higher dose of 2000 mg/kg (*n* = 6). For the 2000 mg/kg dose, the administration was divided into two 1000 mg/kg doses with a period of 4 hours between each administration. Similarly, mice were closely observed for the first 24 h and monitored for 14 days. In addition, a group of mice (*n* = 6) that received only the vehicle orally was used as the control. At the end of the observation time, mice were euthanized with sodium pentobarbital (150 mg/kg). A dissection was performed, and blood was obtained by intracardiac puncture. The liver, kidneys, and brain were then obtained and fixed in 4% formaldehyde for 48 h.

### 2.11. Histopathology Analysis

Tissues were dehydrated using increasing concentrations of ethanol (70%, 80%, 90%, and 100%), cleared with xylene, and then embedded in paraffin. Subsequently, 7 *μ*m sections were obtained using a microtome (ECOSHEL 335), stained with hematoxylin and eosin (H&E), and observed and photographed under an inverted microscope (OPTIKA® IM-3 model) using OPTIKA® ProView x64 version software.

### 2.12. Statistical Analysis

Data were processed using the GraphPad Prism statistical program version 6.01 (San Diego, CA, USA). For the toxicity in brine shrimp, a one-way ANOVA with Tukey's post hoc test was used. For the hemolysis assays and the weights obtained in the acute oral toxicity, a two-way ANOVA followed by Tukey's multiple comparison test was applied. The results were expressed in mean ± standard error of the mean (SEM), and a *p* value of ≤0.05 was considered statistically significant.

## 3. Results

### 3.1. Polyphenol Profile of *F. cernua*

The quantification of hydrolyzable and condensed polyphenols, alongside flavonoids, was conducted in a polyphenolic-enriched extract obtained after AmberLite ion-exchange chromatography. Results indicated a higher concentration of hydrolyzable polyphenols compared to condensed tannins. In addition, the extract displayed a flavonoid content of 124.8 ± 6.9 mg CE/g (Table 1).

Furthermore, the polyphenolic compounds present in the *F. cernua* hydroethanolic leaves extract were identified using HPLC/MS, revealing mainly flavonoids such as flavonols, flavones, and anthocyanins, as well as hydroxycinnamic and methoxycinnamic acids (Table 2 and Supplementary Figure 1). The major compounds identified included apigenin arabinoside glucose (25.69%); 1,3-dicaffeoylquinic, 1-caffeoylquinic acid, and Jaceidin 4′-O-glucuronide (17.56%); and 1, 5-dicaffeoylquinic acid and 3-caffeoylquinic acid (11.52%). Other significant compounds were apigenin 6,8-di-C-glucoside (7.64%) and apigenin (6.36%). These compounds together account for a substantial proportion of the total polyphenolic content, highlighting the richness of the extract in bioactive flavonoids and hydroxycinnamic acids.

### 3.2. Toxicity in Brine Shrimp

**Figure 2:**
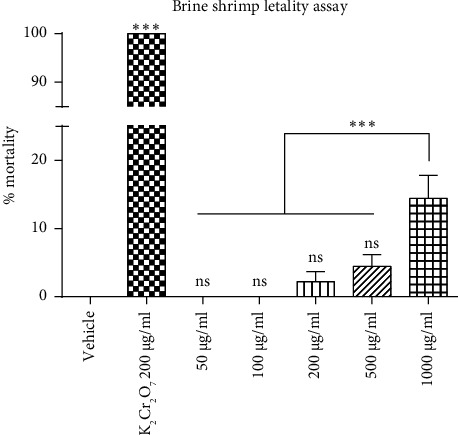
Effect of polyphenolic extract of *F. cernua* on the mortality of brine shrimp. The different treatments are compared against the vehicle. Also, there is a significant difference between the 1000 *μ*g/ml compared to the other doses. Ten brine shrimp per group were used in triplicate, and the experiment was conducted in three independent assays. Statistical analysis was performed using ANOVA followed by Tukey's post hoc test. Data are presented as mean ± SEM. ns: not significant. ^*∗∗∗*^*p* ≤ 0.001.

To evaluate the potential toxicity of *F. cernua* polyphenols, the lethality assay was performed in brine shrimp (Figure 2). The vehicle used to dissolve the polyphenols did not affect the viability of brine shrimp, while the positive control (K_2_Cr_2_O_7_) resulted in a 100% mortality rate. The toxicity tests in brine shrimp revealed that the polyphenolic extract of *F. cernua* exhibited a maximum mortality percentage of 14.4 ± 3.4 at the highest concentration of 1000 *μ*g/ml used in the test, which was significantly different from the other concentrations (*p* ≤ 0.001). Concentrations of 50 and 100 *μ*g/ml did not cause the death of any brine shrimp nauplii. Concentrations of 200 *μ*g/ml and 500 *μ*g/ml resulted in a mortality rate of 2.2 ± 1.5% and 4.4 ± 1.8%, respectively. In addition, all concentrations of the *F. cernua* extract used showed a lower % mortality compared to the positive control (*p* ≤ 0.001).

### 3.3. Hemolysis Assay

**Figure 3:**
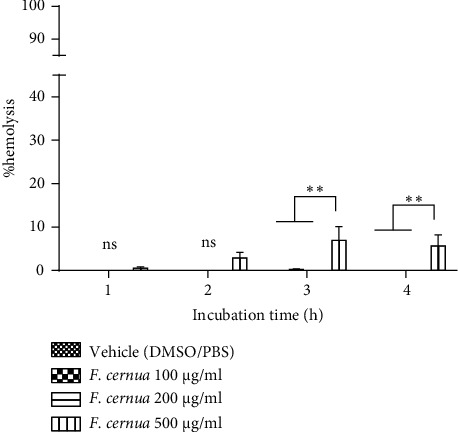
Hemolysis of mice' red blood cells treated with the polyphenolic extract of *F. cernua* dissolved in DMSO: PBS (25 : 75) at different concentrations over various incubation times. No significant hemolysis was observed at 1 and 2 hours of incubation compared to the vehicle. However, significant hemolysis (^*∗∗*^*p* ≤ 0.01) was observed at 3 and 4 hours of incubation with the concentration of 500 *μ*g/ml. Each treatment was performed in triplicate in two independent experiments. Statistical analysis was performed using a two-way ANOVA followed by Tukey's post hoc test. Data are presented as mean ± SEM. ns: not significant. ^*∗∗*^*p* ≤ 0.01.

In the hemolysis assay using mouse and human erythrocytes, various vehicles were tested to dissolve *F. cernua* polyphenol powders. The combination of Tween 80, DMSO, and PBS caused lysis of mouse erythrocytes at all incubation times in a similar way to the positive control used (data not shown). Consequently, a solution of *F. cernua* extract dissolved in a 25 : 75 ratio of DMSO and PBS was used, showing no hemolysis during any incubation period. Concentrations of 100 and 200 *μ*g/ml caused no hemolysis in mouse erythrocytes at any time. However, at 500 *μ*g/ml, hemolysis occurred after the third hour compared to the other concentrations (*p* ≤ 0.05), reaching a hemolysis rate of 7.0 ± 3.1% in the third hour and 5.7 ± 2.5% in the fourth hour (Figure 3).

**Figure 4:**
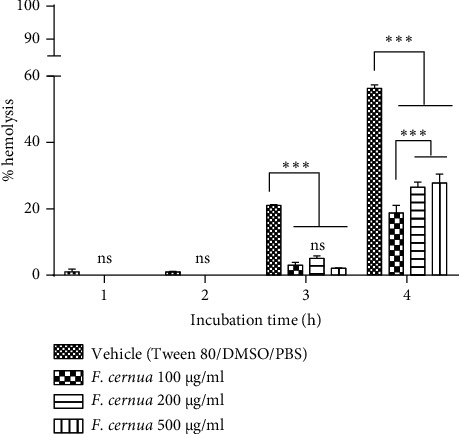
Hemolysis of human red blood cells treated with the polyphenolic extract of *F. cernua* dissolved in Tween 80: DMSO: PBS (5 : 5 : 90) at different concentrations over various incubation times. No significant hemolysis was observed at 1 and 2 hours of incubation compared to the vehicle. However, the vehicle itself caused significant hemolysis starting at 3 hours of incubation. The extracts showed a cytoprotective effect by reducing the hemolysis induced by the vehicle, with this effect being more noticeable at the fourth hour of incubation at the concentration of 100 *μ*g/ml compared to the other concentrations used. Each treatment was performed in triplicate in two independent experiments. Statistical analysis was performed using a two-way ANOVA followed by Tukey's post hoc test. Data are presented as mean ± SEM. ns: not significant. ^*∗∗∗*^*p* ≤ 0.001.

In hemolysis tests with human erythrocytes, using polyphenolic extract of *F. cernua* diluted in Tween 80, DMSO, and PBS (Figure 4), no hemolysis was observed during the first two hours of incubation at any concentration evaluated. In the third hour, concentrations of 100, 200, and 500 *μ*g/ml showed slight hemolysis (3.1 ± 0.8, 5.1 ± 0.8, and 2.1 ± 0.1, respectively), without significant differences. However, in the fourth hour, the percentages of hemolysis notably increased to 18.8 ± 2.2, 26.6 ± 1.5, and 27.8 ± 2.7 for concentrations of 100, 200, and 500 *μ*g/ml, respectively. The concentration of 100 *μ*g/ml exhibited a significant difference compared to 200 and 500 *μ*g/ml (*p* ≤ 0.001).

The comparison between the polyphenolic extract of *F. cernua* and its vehicle revealed a cytoprotective effect on human erythrocytes, as evidenced by a lower level of hemolysis compared to the vehicle. In the first two hours of incubation, the vehicle (Tween 80, DMSO, and PBS) did not induce hemolysis in red blood cells. However, in the third hour, a significant increase in hemolysis was observed in vehicle-treated erythrocytes (21.0 ± 0.3%) compared to those treated with the polyphenolic extract of *F. cernua* dissolved in the vehicle (*p* ≤ 0.001). By the fourth hour, hemolysis induced by the vehicle notably increased to 56.4 ± 1.1%, with a significant difference from erythrocytes treated with the polyphenols (*p* ≤ 0.001). Among the extract concentrations, the 100 *μ*g/ml concentration showed the lowest percentage of hemolysis compared to the others (*p* ≤ 0.001).

**Figure 5:**
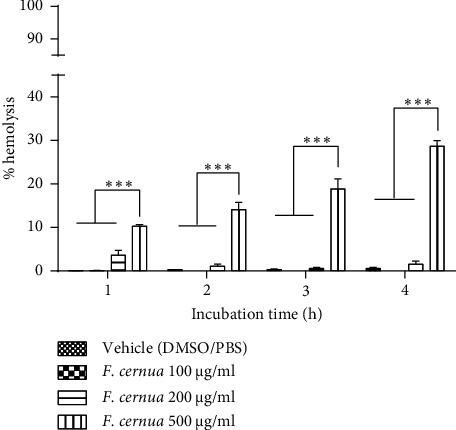
Hemolysis of human red blood cells treated with the polyphenolic extract of *F. cernua* dissolved in DMSO: PBS (25 : 75) at different concentrations over various incubation times. The concentration of 500 *µ*g/ml showed a significant difference in the % hemolysis compared to the other treatments at all incubation times. Each treatment was performed in triplicate in two independent experiments. Statistical analysis was performed using a two-way ANOVA followed by Tukey's post hoc test. Data are presented as mean ± SEM. ns: not significant. ^*∗∗∗*^*p* ≤ 0.001.

The evaluation of % of hemolysis in human erythrocytes using the extract of *F. cernua* dissolved in the vehicle constituted by DMSO: PBS (25 : 75) showed that concentrations of 100 and 200 *μ*g/ml maintained hemolysis values below 5% during the four hours of incubation (Figure 5). However, at 500 *μ*g/ml, a hemolytic effect was observed, escalating over time, and reaching 28.7 ± 1.2% at the fourth hour (*p* ≤ 0.001). These results indicate that polyphenolic extract of *F. cernua* at 100 and 200 *μ*g/ml did not induce significant hemolysis on human erythrocytes. Nonetheless, the concentration of 500 *μ*g/ml exhibited a dose-dependent hemolytic effect over time.

### 3.4. Acute Oral Toxicity

The acute oral toxicity test was performed following OECD 423 guidelines. The oral administration of polyphenolic extract of *F. cernua* at doses of 300 and 2000 mg/kg did not result in mortality in mice. However, signs of toxicity were observed within the first 24 h after dosing. At 300 mg/kg, mice exhibited lethargy and piloerection for 30 min after dosing but showed normal behavior thereafter for the next 24 h and for the entire 14 days of the test.

**Figure 6:**
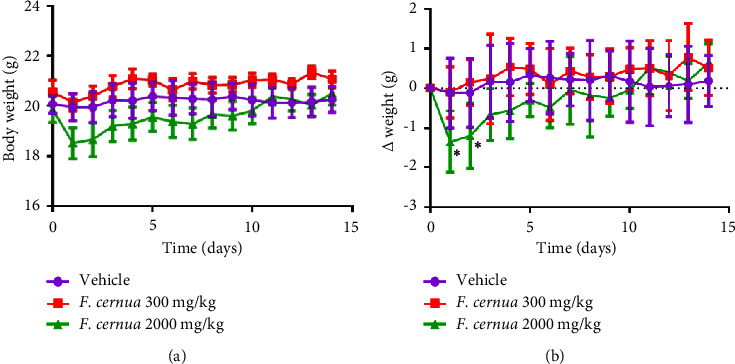
Weight of the mice subjected to the acute oral toxicity test. (a) Weight of the mice at different times after administration of polyphenolic extract of *F. cernua.* (b) Delta weight compared to the initial weight. *N* = 6 mice per group. Statistical analysis was performed using a two-way ANOVA followed by Tukey's post hoc test. Data are presented as mean ± SEM. ns: not significant. ^*∗*^*p* ≤ 0.05.

At the dose of 2000 mg/kg, mice exhibited lethargy, drowsiness, piloerection, reduced appetite, rapid breathing, and hunched posture during the first two hours after extract administration. The frequencies of occurrence of the signs in each experimental group are shown in Supplementary Table 1. Nevertheless, they gradually improved and showed normal behavior after 24 h of administration, which was maintained during the 14-day observation period. The results showed a weight decline in mice administered 2000 mg/kg within the initial 48 hours, but gradually regained their initial weight over the subsequent days (Figure 6(a)). When comparing group weights before and after administration (Figure 6(b)), a significant initial decrease was observed in the 2000 mg/kg group compared to the control (*p* ≤ 0.05) during the first two days after administration.

### 3.5. Histopathological Analysis of Liver, Kidney, and Brain

**Figure 7:**
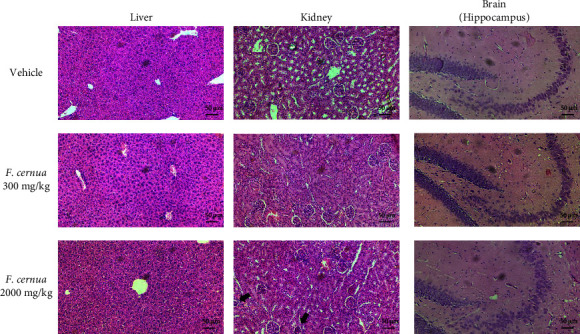
Photomicrographs of hematoxylin and eosin (H&E)-stained sections of the liver, kidney, and brain from mice treated with vehicle and the polyphenolic extract of *F. cernua* (300 and 2000 mg/kg). Black arrows indicate shrunken glomeruli in mice that received the 2000 mg/kg dose. Scale bar: 50 *μ*m.

Histological examination of the liver, kidney, and brain tissues of mice was conducted to assess any potential alterations caused by the administration of *F. cernua* extract (Figure 7). Liver tissues from all experimental groups, including the vehicle group and both doses of the *F. cernua* extract, exhibited a normal hepatocellular architecture. Observations showed intact central veins and trabeculae without any histological abnormalities detected in liver tissue. Regarding the kidneys, both the vehicle group and the 300 mg/kg dose group of *F. cernua* extract displayed normal glomerular and renal tubular architecture. However, in the group administered with the higher dose of 2000 mg/kg of polyphenolic extract, a reduction in glomerular size and shrinkage was observed, indicating potential mild renal alterations at this higher dose. Analysis of brain tissue from all experimental groups did not show noticeable histological alterations in the hippocampus, cortex, and cerebellum region (Supplementary Figure 2). In summary, histopathological evaluation of liver, kidney, and brain tissues suggests that the administration of *F. cernua* polyphenolic extract, particularly at the higher dose, may induce mild changes in the kidneys without affecting liver and brain tissues.

## 4. Discussion

In regions with restricted healthcare access, such as remote arid zones, communities have traditionally relied on herbal remedies to address diverse diseases [23]. *Flourensia cernua*, a plant thriving in the semidesert areas of Chihuahua, is commonly used in leaf infusions or decoctions to alleviate gastrointestinal ailments [24]. Despite its traditional usage, comprehensive data on the compounds within its leaves and the potential associated toxicity after consumption remain limited.

In this study, we obtained a hydroethanolic extract of *F. cernua* using emerging ultrasound-microwave techniques, followed by purification of polyphenols via ion-exchange chromatography. The polyphenolic extract showed a notably higher content of hydrolyzable polyphenols (257.7 ± 8.8 mg GAE/g) compared to condensed polyphenols (100.2 ± 13.2 mg CE/g). This observation aligns with previous findings by Estell et al. [25], who also highlighted a predominance of hydrolyzable polyphenols in the plant compared to condensed polyphenols. In other studies, the hydrolyzable polyphenols of *F. cernua* were quantified obtaining values of 301 ± 0.2 mg GAE/g and 53.07 mg GAE/g by the Soxhlet extraction method [5, 12]. Different extraction methods, solvents, soil conditions, nutrient availability, temperature, light intensity, harvest season, and degree of plant maturation can contribute to variations in polyphenol content [26, 27]. The extract is rich in flavonoids (124.8 ± 6.9 mg CE/g), which is in agreement with a study reporting quercetin equivalent concentration within the range of 258.22–133.77 *μ*g/ml [8], emphasizing, *F. cernua* leaves as a rich source of flavonoid-like compounds. The HPLC/MS analysis identified 20 distinct compounds in the *F. cernua* extract, predominantly belonging to flavonoids and hydroxycinnamic acids. Flavonoids such as flavones, flavanones, and flavonols constitute the primary polyphenolic components of the Asteraceae family [9, 28]. Previous studies have also identified compounds present in this plant. Álvarez-Perez et al. [10] and Aranda-Ledesma et al. [8] identified 15 and 18 different polyphenolic compounds, respectively, with the major compounds being apigenin-6-C-glucosyl-8-C-arabinoside and luteolin 7-O-rutinoside. In the present study, apigenin and its glycosides were identified as the primary compounds in the polyphenolic extract, which is consistent with prior research findings. The abundance of flavones and other flavonoids in *F. cernua* may be attributed to their role as UVB protectors in plants, as they absorb light within the 280–315 nm range. Due to the semidesert habitat and high UVB exposure of *F. cernua*, a high polyphenol content, especially flavones, is expected [29, 30]. Apigenin, a widespread flavonoid in plants, is commonly found in the Asteraceae family, including genera such as *Artemisia*, *Achillea*, *Matricaria*, and *Tanacetum* [31]. The findings of this study highlight *F. cernua* as a significant source of apigenin and other flavonoids, suggesting potential medicinal implications and health benefits. However, further research is essential to explore the pharmacological activities and potential therapeutic applications of these polyphenolic compounds. In addition, comprehensive investigations are needed to understand their pharmacological properties and safety aspects, particularly concerning potential therapeutic applications.

In addition to exploring the therapeutic potential of medicinal plants, assessing their potential toxic effects is also essential, as the negative impact on public health may remain unknown. In the present study, the acute toxicity of a polyphenolic extract of *F. cernua* was evaluated through both *in vitro* and *in vivo* tests. Among these tests, the *Artemia* toxicity test was chosen due to the brine shrimp's sensitivity to toxic compounds, making it an excellent model for initial toxicity screening [32]. This assay offers numerous advantages, including cost-effectiveness, easy availability of commercial cysts, uniformity of the nauplii population after hatching, straightforward laboratory handling and maintenance, and the ability to perform assays in microplates [33]. In addition, *Artemia* nauplii do not require feeding during the initial 72 h after hatching, making them highly sensitive to substances they come into contact with during this period [34]. The toxicity test with *Artemia* nauplii revealed that concentrations of 50 and 100 *μ*g/ml of *F. cernua* extract were harmless to these invertebrates after 24 h of incubation. Furthermore, at a concentration of 1000 *μ*g/ml, the maximum mortality observed was 14.4%, indicating that the lethal concentration of 50 (LC_50_) is greater than 1000 *μ*g/ml. An extract is generally considered nontoxic if its LC_50_ value exceeds 1000 *μ*g/ml [35]. Therefore, the polyphenolic extract of *F. cernua* obtained in this study is considered nontoxic to *Artemia salina*. Considering the lack of existing reports on the toxicity of *F. cernua* toward *Artemia* or other aquatic invertebrates, the importance of investigating the toxicity of the extract in these organisms is highlighted. This is especially critical in the context of potential applications of this plant, such as its use as a biocide [10, 18]. Understanding the impact of *F. cernua* extract on aquatic organisms can help ensure its safe utilization in various applications, including agriculture.

Another *in vitro* test conducted was the hemolysis assay, which assesses the cytotoxicity of compounds or extracts by measuring erythrocyte rupture and hemoglobin release using spectrophotometry [36]. This assay directly evaluates the potential damage that an extract may cause to the erythrocyte cell membrane. While this assay does not replicate the complex interactions within the human body, it provides valuable insight into the extract's potential impact on cell membranes [37]. For this experiment, mouse and human erythrocytes were used along with two different vehicles for dissolving the extracts. In the case of mouse erythrocytes, the vehicle composed of Tween 80, DMSO, and PBS resulted in erythrocyte lysis from the first hour. Saebo et al. reported that Tween 20 induces erythrocyte lysis at concentrations of ≥0.01%. Notably, human erythrocytes exhibited two times less lysis compared to mouse and rat erythrocytes [38]. In this study, a final concentration of 0.1% Tween 80 was used, causing erythrocyte lysis; hence, a vehicle without Tween 80 was employed. The DMSO: PBS vehicle did not cause any erythrocyte lysis during the 4h incubation period. Moreover, at a concentration of 500 *μ*g/ml, the polyphenolic extract of *F. cernua* exhibited hemolysis after 3 h of incubation, reaching a maximum value of 7.0 ± 3.1%, which is considered nontoxic [39]. Regarding the hemolytic activity of the *F. cernua* extract on human erythrocytes, two vehicles were also used. The vehicle composed of Tween 80, DMSO, and PBS resulted in hemolysis after the third hour of incubation, reaching a maximum hemolysis percentage of 56.4 ± 1.1 at 4 h of incubation. The hemolytic effect was attributed to Tween 80, a nonionic detergent [40]. Detergents possess a similar structure to the lipids in the plasma membrane, allowing them to penetrate the membrane and cause a reorganization of lipids and membrane proteins, ultimately leading to changes in erythrocyte morphology and lysis [41]. Interestingly, the *F. cernua* extract showed lower hemolysis compared to the vehicle, possibly due to the presence of polyphenols, known for their cytoprotective effect on erythrocytes against oxidizing agents and hypotonic conditions [42, 43]. The potential mechanism involves the polyphenols of the extract of *F. cernua* and their interaction with the polar heads of the membrane lipids, forming a barrier that prevents the detergent from interacting with the plasmatic membrane [44]. In addition, the cytoprotective effect of apigenin on human erythrocytes subjected to oxidative damage by H_2_O_2_ has been reported. The proposed mechanism is that apigenin could restore cell surface morphology by protecting the sulfhydryl content in the red cell membrane protein [45]. This cytoprotective effect was more noticeable at a concentration of 100 *μ*g/ml, as higher concentrations resulted in increased hemolysis. In addition, the DMSO: PBS vehicle did not cause any damage to erythrocytes at any incubation hour. Similarly, the polyphenolic extract of *F. cernua* caused hemolysis only at a concentration of 500 *μ*g/ml, reaching a value of 28.7 ± 1.2% at the fourth hour. This percentage, considered slightly toxic, was observed during the last hour of incubation [39]. Extending the incubation time aimed to confirm that polyphenolic compounds would not affect the erythrocyte membrane, as certain compounds might require more time to interact and induce hemolysis [38]. This is evident in the results since the analysis after only one hour of incubation would not have shown hemolysis in any of the treatments with the *F. cernua* extract. These results highlight the potential therapeutic value of the extract of *F. cernua* due to its cytoprotective properties and low hemolytic activity.

In the acute oral toxicity test, no deaths occurred among tested animals at doses of 300 and 2000 mg/kg. According to the OECD criteria, the LD_50_ of the *F. cernua* extract is >2000–5000 mg/kg, categorized as category 5, and can be considered safe [46]. However, some signs that could indicate slight intoxication, such as lethargy, drowsiness, and piloerection, were observed within the first hours after administering the *F. cernua* extract. In addition, mice showed significant weight loss during the initial two days following the administration of the extract at a dose of 2000 mg/kg. These findings align with a study by Zavala et al. [16], where an ethanolic extract at 2000 mg/kg did not cause death in Wistar rats. However, slight signs of toxicity, such as lethargy and drowsiness, were observed, although no differences were found in terms of body weight. In contrast, in the present study, a decrease in animal weight was observed at the dose of 2000 mg/kg. Furthermore, the histopathological analysis revealed no alterations in liver and brain tissue. However, in relation to the kidney, observations showed some shrunken and smaller glomeruli, indicating possible kidney damage after administration of the 2000 mg/kg dose. It is noteworthy that the toxicity of *F. cernua* appears to vary depending on the part of the plant, as dried fruit has been reported to be toxic to cattle, causing inflammation, ulcerations, and perforation of the gastrointestinal tract within the first 24 h after consumption [47]. In contrast, leaf extracts do not appear to have any lethal effect after acute consumption. However, there are reports suggesting that prolonged consumption may negatively impact liver function [15]. Therefore, one of the perspectives is to evaluate the subacute and chronic effects that consumption of *F. cernua* extracts could have on liver and kidney function to determine whether prolonged consumption is safe for consumers.

## 5. Conclusions

In this study, a polyphenolic extract of *Flourensia cernua* was obtained using a novel ultrasound and microwave extraction method, followed by purification through ion-exchange chromatography. Analysis revealed its richness in flavonoids and hydroxycinnamic acids, with apigenin and its glycosides being the most abundant compounds. The extract showed no toxicity to *Artemia salina* and a low hemolytic effect on erythrocytes. In the acute oral toxicity test in mice, doses of 300 mg/kg and 2000 mg/kg were administered, and neither dose resulted in mortality among the animals. These findings place the extract in category 5 according to the OECD standards, indicating that it is safe at tested concentrations. However, signs of potential toxicity, such as weight loss and kidney structural alterations, were observed at the highest dose tested. Therefore, further research is crucial to assess the safety profile of the extract under chronic exposure conditions and to evaluate its impact on liver and kidney functions following oral administration.

## Figures and Tables

**Table 1:** Polyphenol content of the polyphenolic-enriched extract of *F. cernua*.

Compound	Amount (mean ± SEM)
Hydrolyzable polyphenols (mg·GAE/g)	257.7 ± 8.8
Condensed tannins (mg·CE/g)	100.2 ± 13.2
Total flavonoids (mg·CE/g)	124.8 ± 6.9

**Table 2:** Main polyphenolic compounds in the polyphenolic extract of *F. cernua*.

	Compound	Family	RT (min)	*m*/*z* ([M-H]^−^)	Area (%)
1	Myricetin	Flavonols	6.415	316.8	1.30
2	Apigenin 6, 8-di-C-glucoside	Flavones	31.171	592.8	7.64
3	Apigenin arabinoside-glucoside	Flavones	33.62	562.9	25.69
4	Chrysoeriol 7-O-(6″-malonyl-glucoside)	Methoxyflavones	37.935	546.9	1.73
5	3,7-Dimethylquercetin	Methoxyflavonols	38.47	328.9	1.61
6	Quercetin	Flavonols	38.747	300.8	1.63
7	Rhoifolin	Flavones	38.805	576.8	2.45
8	1-Caffeoylquinic acid	Hydroxycinnamic acids	41.59	352.9	17.56
9	1,3-Dicaffeoylquinic acid	Hydroxycinnamic acids	41.59	514.8	17.56
10	Jaceidin 4′-O-glucuronide	Methoxyflavonols	41.59	536.8	17.56
11	1,5-Dicaffeoylquinic acid	Hydroxycinnamic acids	43.183	514.9	11.52
12	3-Caffeoylquinic acid	Hydroxycinnamic acids	43.183	352.9	11.52
13	6,8-Dihydroxykaempferol	Flavonols	45.589	316.9	3.73
14	Malvidin 3-O-galactoside	Anthocyanins	46.48	528.9	3.37
15	3-Feruloylquinic acid	Methoxycinnamic acids	46.48	366.9	3.37
16	Malvidin 3-O-galactoside	Anthocyanins	47.083	528.9	3.01
17	Cirsimaritin	Methoxyflavones	48.62	313	5.80
18	Protocatechuic acid 4-O-glucoside	Hydroxybenzoic acids	52.185	314.9	4.55
19	Feruloyltartaric acid	Methoxycinnamic acids	53.595	325	2.05
20	Apigenin	Flavones	54.018	268.9	6.36

RT, retention time; *m*/*z*, mass-to-charge ratio.

## Data Availability

The data used to support the findings of this study are available from the corresponding author upon request.

## Supplementary Materials

Supplementary Materials Supplementary Table 1: simulated seawater recipe for 1 L. Supplementary Table 2: frequency of toxicity signs during the first 4 hours observed in mice treated with *F. cernua* polyphenolic extract at doses of 300 mg/kg and 2000 mg/kg. Supplementary Figure 1: chromatogram of *F. cernua* polyphenolic extract sample. Supplementary Figure 2: photomicrographs of hematoxylin and eosin (H&E)-stained section of cerebral cortical and cerebellum from mice treated with vehicle and the polyphenolic extract of *F. cernua* (300 and 2000 mg/kg). Scale bar: 50 *μ*m.
