# Supplementary material for: Toxicity Evaluation of a Polyphenolic Extract from Flourensia cernua DC through Artemia Lethality Assay, Hemolytic Activity, and Acute Oral Test
Source: J Toxicol. 2024 Aug 9;2024:2970470. doi: 10.1155/2024/2970470 (PMC11329308; doi:10.1155/2024/2970470)
Supplement: Supplementary Materials — Supplementary Table 1: simulated seawater recipe for 1 L. Supplementary Table 2: frequency of toxicity signs during the first 4 hours observed in mice treated with F. cernua polyphenolic extract at doses of 300 mg/kg and 2000 mg/kg. Supplementary Figure 1: chromatogram of F. cernua polyphenolic extract sample. Supplementary Figure 2: photomicrographs of hematoxylin and eosin (H&E)-stained section of cerebral cortical and cerebellum from mice treated with vehicle and the polyphenolic extract of F. cernua (300 and 2000 mg/kg). Scale bar: 50 μm. [file 2970470.f1.zip › Supplementary Table 1.docx]

**Supplementary Table 1.** Simulated sea water recipe for 1 L.

| Reactive | Quantity (g) |
| --- | --- |
| NaCl | 26.4 |
| KCl | 0.84 |
| CaCl_2_·2H_2_O | 1.67 |
| MgCl_2_·6H_2_O | 4.60 |
| MgSO_4_·7H_2_O | 5.58 |
| NaHCO_3_ | 0.17 |
| H_3_BO_3_ | 0.03 |
