# Supplementary material for: Toxicity Evaluation of a Polyphenolic Extract from Flourensia cernua DC through Artemia Lethality Assay, Hemolytic Activity, and Acute Oral Test
Source: J Toxicol. 2024 Aug 9;2024:2970470. doi: 10.1155/2024/2970470 (PMC11329308; doi:10.1155/2024/2970470)
Supplement: Supplementary Materials — Supplementary Table 1: simulated seawater recipe for 1 L. Supplementary Table 2: frequency of toxicity signs during the first 4 hours observed in mice treated with F. cernua polyphenolic extract at doses of 300 mg/kg and 2000 mg/kg. Supplementary Figure 1: chromatogram of F. cernua polyphenolic extract sample. Supplementary Figure 2: photomicrographs of hematoxylin and eosin (H&E)-stained section of cerebral cortical and cerebellum from mice treated with vehicle and the polyphenolic extract of F. cernua (300 and 2000 mg/kg). Scale bar: 50 μm. [file 2970470.f1.zip › Supplementary Table 2.docx]

**Supplementary Table 2.** Frequency of toxicity signs during the first 4 hours observed in mice treated with *F. cernua* polyphenolic extract at doses of 300 mg/kg and 2000 mg/kg.

| **Parameters** | **Vehicle** | ***F. cernua* 300 mg/kg** | ***F. cernua* 2000 mg/kg** |
| --- | --- | --- | --- |
| Lethargy | 0/6 | 3/6 | 6/6 |
| Piloerection | 0/6 | 2/6 | 4/6 |
| Drowsiness | 0/6 | 3/6 | 6/6 |
| Reduced appetite | 0/6 | 0/6 | 2/6 |
| Rapid breathing | 0/6 | 0/6 | 4/6 |
| Hunched posture | 0/6 | 0/6 | 2/6 |
| Diarrhea | 0/6 | 0/6 | 0/6 |
| Deaths | 0/6 | 0/6 | 0/6 |

In the case of the 2000 mg/kg dose, parameters were evaluated at the end of the administration of the two 1000 mg/kg doses.
