# Supplementary figures and images for: Toxicity Evaluation of a Polyphenolic Extract from Flourensia cernua DC through Artemia Lethality Assay, Hemolytic Activity, and Acute Oral Test
Source: J Toxicol. 2024 Aug 9;2024:2970470. doi: 10.1155/2024/2970470 (PMC11329308; doi:10.1155/2024/2970470)

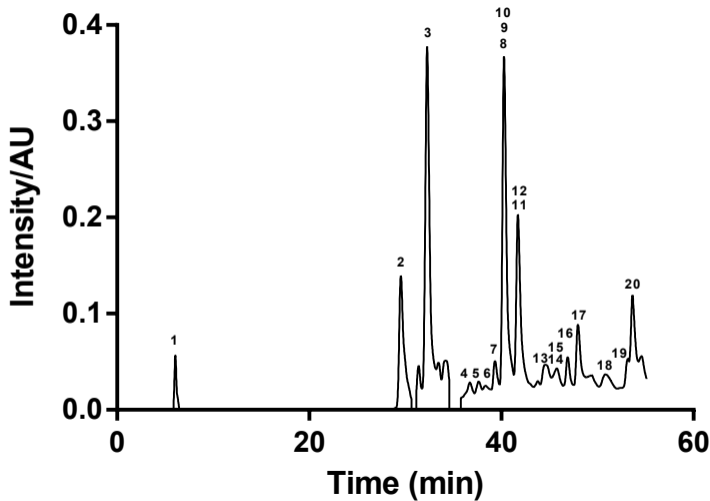

Supplement: Supplementary Materials — Supplementary Table 1: simulated seawater recipe for 1 L. Supplementary Table 2: frequency of toxicity signs during the first 4 hours observed in mice treated with F. cernua polyphenolic extract at doses of 300 mg/kg and 2000 mg/kg. Supplementary Figure 1: chromatogram of F. cernua polyphenolic extract sample. Supplementary Figure 2: photomicrographs of hematoxylin and eosin (H&E)-stained section of cerebral cortical and cerebellum from mice treated with vehicle and the polyphenolic extract of F. cernua (300 and 2000 mg/kg). Scale bar: 50 μm. [file 2970470.f1.zip › Supplementary Figure 1.pdf]

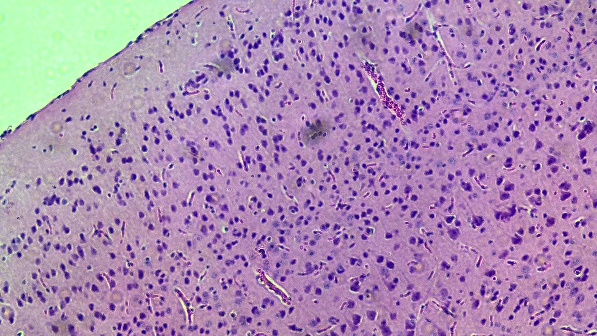

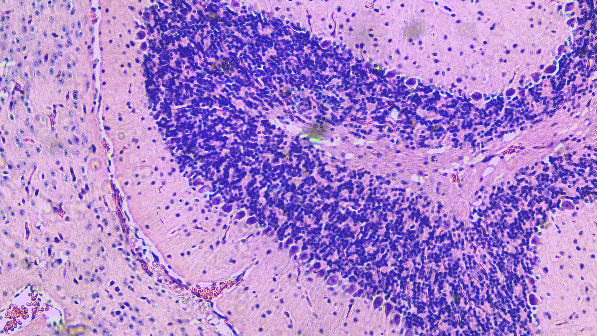

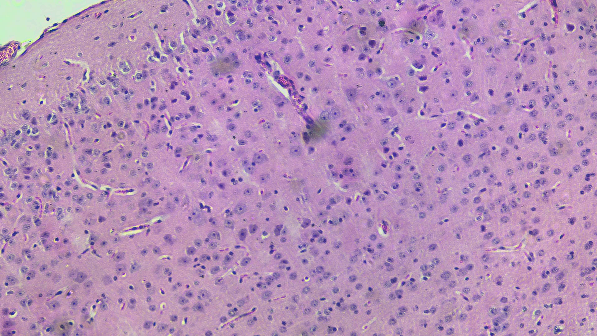

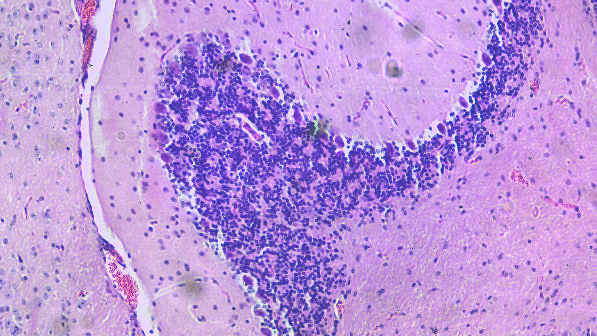

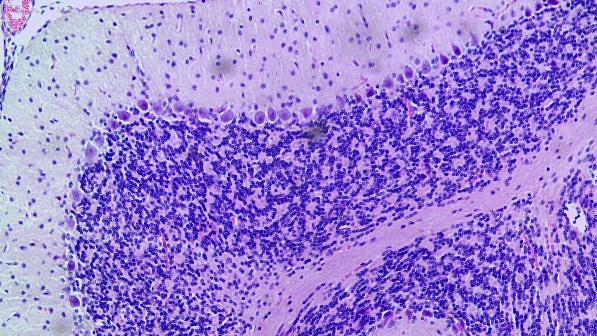

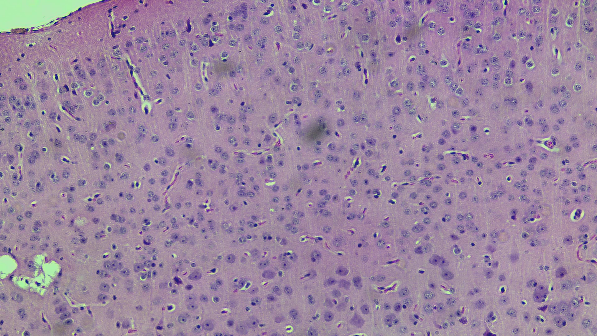


**Cerebral cortical**

**Cerebellum**

**Vehicle**

***F. cernua 300 mg/kg***

***F. cernua 2000 mg/kg***

**50 µm**

**50 µm**

**50 µm**

**50 µm**

**50 µm**

**50 µm**

Supplement: Supplementary Materials — Supplementary Table 1: simulated seawater recipe for 1 L. Supplementary Table 2: frequency of toxicity signs during the first 4 hours observed in mice treated with F. cernua polyphenolic extract at doses of 300 mg/kg and 2000 mg/kg. Supplementary Figure 1: chromatogram of F. cernua polyphenolic extract sample. Supplementary Figure 2: photomicrographs of hematoxylin and eosin (H&E)-stained section of cerebral cortical and cerebellum from mice treated with vehicle and the polyphenolic extract of F. cernua (300 and 2000 mg/kg). Scale bar: 50 μm. [file 2970470.f1.zip › Supplementary Figure 2.docx]
